# From the model of integral attention to the creation of centers of excellence in rheumatoid arthritis

**DOI:** 10.1007/s10067-015-3017-8

**Published:** 2015-07-26

**Authors:** Pedro Santos-Moreno, Oswaldo Castañeda, Boris Garro, Dennis Flores, Guillermo Sánchez, Carlos Castro

**Affiliations:** BIOMAB: Rheumatoid Arthritis Center, Calle 48 N 13-86, Bogotá, Colombia; Clinica Anglo-Americana, Lima, Peru; Clinica Ricardo Palma and Osteoporosis Center, Lima, Peru; Salvadorean Social Security Institute, San Salvador, El Salvador; SIIES research and education in health, Bogotá, Colombia

**Keywords:** Centers of excellence, Competitiveness, Comprehensive patient care, Efficiency, Health care quality, Rheumatoid arthritis

## Abstract

For the Pan American Health Organization (PAHO), the care of patients with chronic diseases currently experiences fragmentation in attention, generating poor performance of health services. Thus, comprehensive health care strategies arise to mitigate these problems; one of them are Centers of Excellence (CoEs), which aim to obtain high quality results in health from the adequate and minimum use of resources. The objective of this study was to describe the history and current context of the CoE in comprehensive care in patients with rheumatoid arthritis (RA). A systematic search of the literature terms (MeSH) was performed. The bases used were PubMed, Ebsco Host, Lilacs, Science Direct, Ovid, and Google (gray literature). The source of the information was evaluated to determine its quality. International standards focus the CoEs starting from comprehensive management of patients with RA and patient volume, continuous improvement, and quality of health care, constituting an interdisciplinary team. The REAL-PANLAR group suggested that the inclusion of the strategy “Treat to Target”, and patient education improves patient conditions and understanding of the disease. RA is a prevalent and costly disease. The creation of comprehensive care centers of the CoE type is an initiative that improves the prognosis of RA. This document aims to encourage rheumatologists and scientific societies to structure CoE in an interdisciplinary endeavor.

## Introduction

For the Pan American Health Organization (PAHO), the care of patients with chronic diseases currently experiences fragmentation in comprehensive care, generating poor performance of health services, and demonstrating difficulties of access, diagnosis, treatment, and management of the patient environment. Additionally, it faces a deficit of technical quality due to the irrational and inefficient use of resources, adding to the negative perception by the users [1]. This scenario is the result of a lack of administrative organization, where the economic aspect has been prioritized, leaving the patient in the background. Because of this, strategies to mitigate these problems that are articulated with elements of public health arise. One of them is creating highly specialized comprehensive care centers which aim to concentrate the population and centralize the management of the disease, which has led to the creation of so-called Centers of Excellence (CoEs), which seek to obtain high quality results from the appropriate and minimal use of resources as part of what Castaño suggested starting from three fundamental pillars: (1) the volume/demand of the specific condition or diagnosis, (2) the culture of continuous improvement, and (3) the quality of the health care professional [2]. This initiative began in the 1990s, due to the shortcomings of health systems, where the monopolization by the supplier does not meet demand, taking into account the geographical distribution of patients (rural and urban areas and the difficulty of access) and coverage throughout the country. In addition to this problem, there is the lack of availability of health professionals to manage chronic diseases [3] that impact the patients’ psychological, social, and economic aspects. The clinical features of rheumatoid arthritis (RA) and its epidemiological behavior justify the need for a management that involves all of the patient’s spheres and their context. It has been estimated that the prevalence of RA in Latin America is between 0.4 and 1.6 %. A study done in Mexico shows that the prevalence in this country is of 1.6 % with a CI of 95 % (between 1.4 and 1.8 %) and that this disease is more frequent in women with a ratio of 3–1 [4, 5]. This condition is associated with emotional disorders due to chronic pain, permanent functional impairment, anatomical deformities, and loss of independence within their environment [6].

The World Health Organization (WHO) suggests that for the proper care of RA, there should be a rheumatologist per 100,000 people, i.e., that about 5000 specialists would be needed for Latin America; however, there are 19 rheumatology societies grouping about 2000 members, showing a problem in diagnosis and treatment for lack of human resources. Additionally, the quality indicators that are evaluated in different models of care are not met fully due to the lack of an organizational structure that allows for a comprehensive approach to patient management [1, 7]. Another key element in the problem identified is the variation in behavior for the treatment of RA, because despite the existence of evidence-based clinical practice guidelines there is no unifying handling compliance, directly affecting the clinical course of the disease [7, 8]. This series of obstacles faced by patients, health professionals, and health services make the argument for structuring organizations capable of focusing on the patients and their condition in order to improve the quality of service delivery as the structuring of CoEs proposes. Therefore, this paper aims to describe the history and current context of Centers of Excellence in comprehensive care in patients with rheumatoid arthritis (RA).

## Methods

**Fig. 1:**
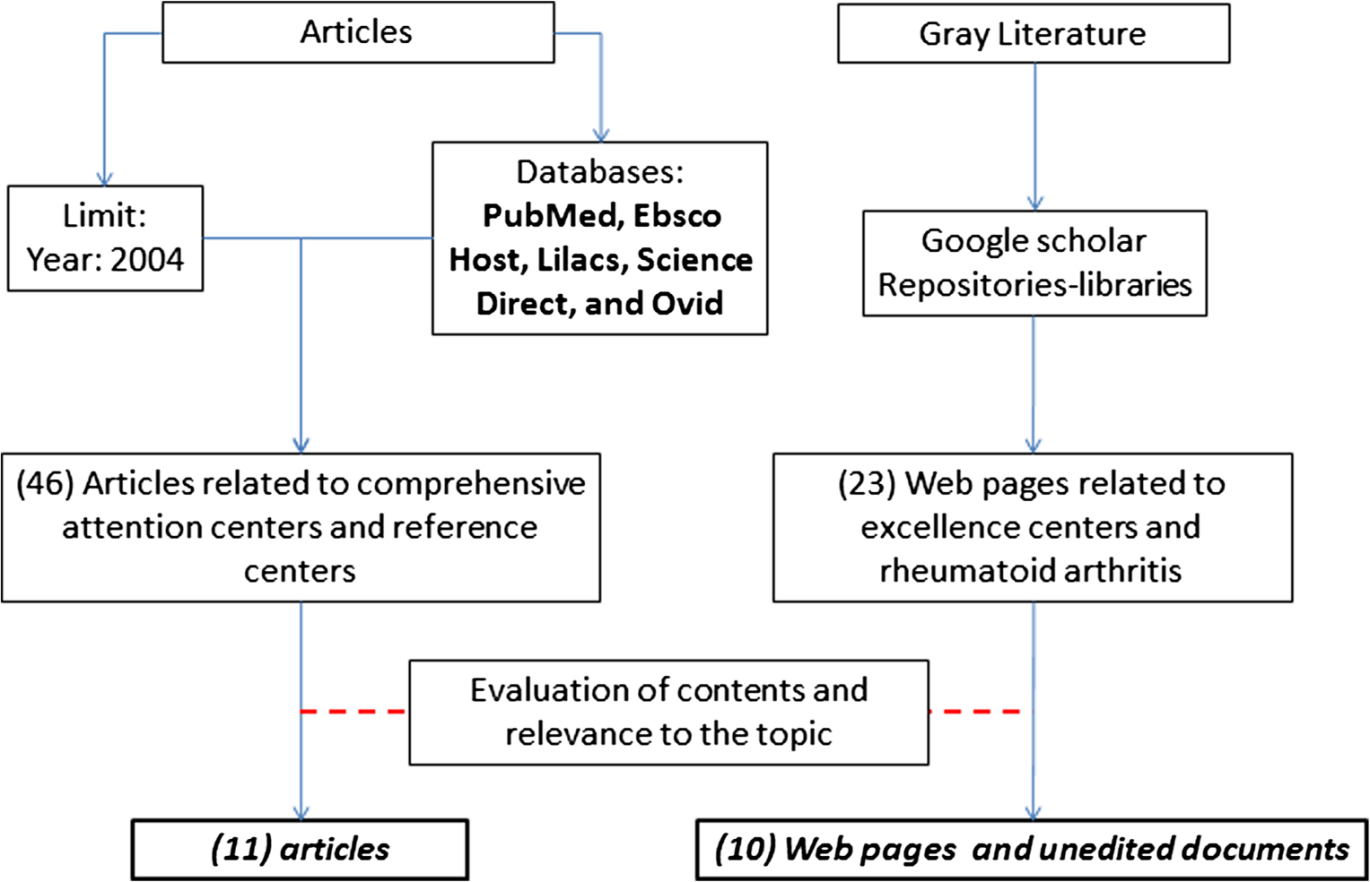
Source of the information

A systematic literature search with the following keywords was performed: rheumatology, quality, efficiency (MeSH), Center of Excellence, comprehensive care, and learning curve, which were combined using Boolean operators AND, OR, and NOT. Search was not limited by year or by language. The databases searched were PubMed, Ebsco Host, Lilacs, Science Direct, and Ovid. Additionally, gray literature was considered using the metasearch engine Google Scholar. The source of the information was evaluated to determine its quality and affiliations of the authors (Fig. 1).

## Results

### Approximation and route for diagnosing of the RA patient

The success of the diagnosis of RA begins with clinical suspicion of the disease in primary care levels, where the identification of patients with symptoms suggestive of RA should be integrated special programs for disease management. In a systematic review, Chan found the path that patients usually follow and the obstacles they face, added to those previously described, until reaching specialized treatment, while at the same time identified the solutions that could help improve the quality of patient care. The same study reported the average time for accessing health services: first moment, the user consults at the first level (4–12 weeks); second moment, the patient is referred to a rheumatologist (2–3 months); and third moment, the patient receives treatment from his or her insurer (2–3 weeks) [8]. On the other hand, Ruiz says that the availability of nurses in a leading RA attention center must be organized as follows: one nurse for every three consulting rooms, an auxiliary nurse for every two consulting rooms, and an administrative for every four doctors. Ruiz also states that a health system should have three beds for RA patients per 100,000 inhabitants considering the statistics described. These parameters are critical to improving health care quality and reduce access times [9, 10].

**Fig. 2:**
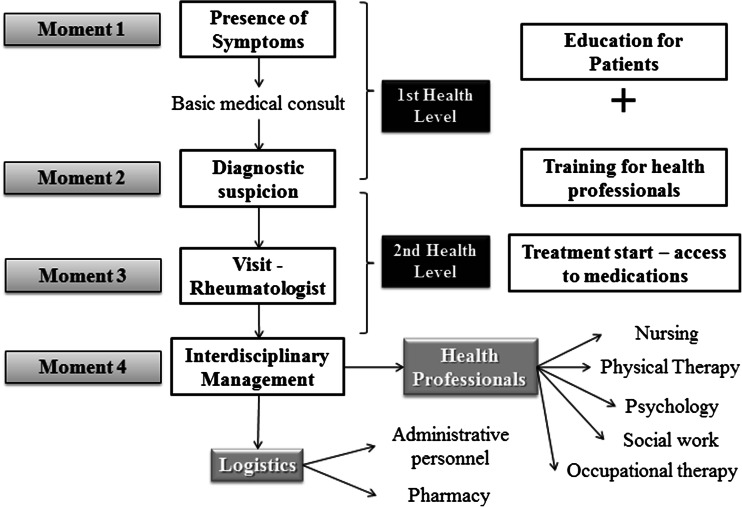
The health system

This procedure is affected by factors inherent in the system and the patient’s difficulties, due to the lack of information or disability that prevents management for the monitoring of the disease (Fig. 2) [11].

Such proposals are the gateway to the formation of interdisciplinary teams for the comprehensive management of AR, allowing the structuring of centers of excellence.

### Centers of excellence

**Fig. 3:**
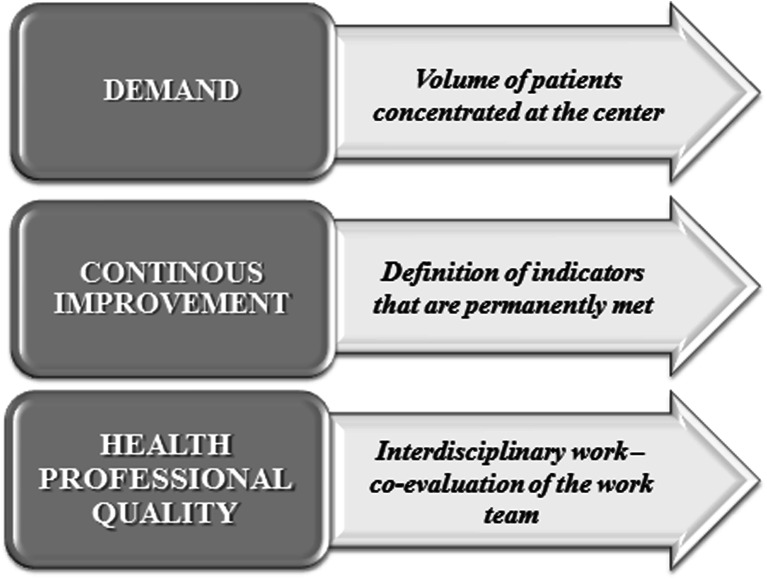
Learning curve for healthcare

According to the above, the CoEs are a sustainable competitive strategy, both locally and internationally, which is understood as a health program that fully complies with indicators of effectiveness and safety at competitive costs in the marketplace. According to Castaño, this theory is based on the learning curve (acquisition of cognitive and manual skills) of health care [2], which leads to the three pillars of structuring a CoE (Fig. 3).

#### Rheumatoid arthritis and pillars of excellence

*“The volume of patients with a specific condition or entity”* includes the first pillar, and where the target population are patients with RA. This pillar aims to ensure a considerable number of patients, allowing the CoE group to concentrate on RA pathology and feed the experience curve for health professionals [12] and ensure comprehensive care. For the organization of the centers, it is important to note that different health systems in each country should collaborate with patient mobility setting aside self-interest of the provider and opening the possibility that the focus is on the patient. Once the logistics to collect and cite patients in a single center are obtained, it must define a program of continuous improvement, quality assurance processes, and procedures in comprehensive care, adding knowledge management, which involves the research and technological innovation, supported by a group of experts which improves clinical and administrative decision-making. Castaño explains this pillar, with the PDVA acronym, where each letter stands for Plan-Do-Verify-Act [2].

The second pillar refers to the concept of *“continuous improvement”*, which is based on all activities that organizations or companies constantly perform to improve quality processes [3] with a competitive approach and improving the patient’s or user’s perceptions. In this vein, the responsibility of the scientific societies is not limited to the organization of conferences and meetings, but are also indirectly responsible for patient care, which must conform to international and local lineaments, which in often are the result of rigorous and systematic processes such as the evidence-based guidelines for clinical practice.

For this reason, the Pan American League of Associations for Rheumatology (PANLAR), currently leads a number of initiatives to establish centers to fully treat patients with RA, for which it assigned the “Real–PANLAR project for the implementation and accreditation of centers of excellence in rheumatoid arthritis throughout Latin America”, project to a group of Latin American rheumatologists who after a thorough bibliographic research, and a Delphi type expert consensus, suggests the following indicators for a center of excellence in RA [7, 13, 14]:

1. Structure indicators: Evaluate the institutional capacity to deliver the expected results, adequate infrastructure, suitable personnel including rheumatologists and other professionals to ensure comprehensive attention, and the existence of complementary resources (e.g., clinical laboratory and imaging).
2. Process indicators: Adherence to management recommendations based on treatment strategy by objectives Treat to Target (T2T), in addition to including educational processes directed to the patient by the interdisciplinary team.
3. Outcome indicators: The achievement of the objectives proposed along the care or comprehensive patient must be evaluated. The progression of the disease, functional disability, and the achievement of remission goals must be quantified using clinimetric scales such as joint counts, DAS28, HAQ, CDAI, and ACR among others.

Furthermore, a conceptually comprehensive quality care means taking into account a number of recommendations that enable better care [7, 13, 14]:

- Committees to define the integrity of treatment, where issues such as disease activity, quality of life, compliance, and adherence to treatment are involved.
- Unification of handling and socialization of clinical practice guidelines according to each country.
- Minimum standard evaluation based on the T2T strategy.
- Defining the processes and procedures for patients to access groups of experts in RA
- Increasing the patients’ involvement in disease management with effective pedagogical activities.
- Constitution of information systems for multi-centric studies nationally and internationally.
- Negotiating with the different health systems the implementation of strategies to reorganize the distribution of patients to different groups or specific centers in attention of RA [7].

Compliance with these parameters strengthens the competitiveness of the centers specialized in RA management, allowing for offering the best service, which must constantly be compared with similar centers. According to Porter, competition in health care has become a noncompetitive monopoly where providers divide the market amongst them, or in the worst cases, are concentrated in a few. This causes costs to be transferred, but not reduced. Currently, the costs are shifted, from supplier to hospital, hospital to doctor, and doctor to patient, without generating a net cost concentrated by pathology or entity, dispersing the impact on the system and encouraging the cheaper and less effective treatments. In an adequate competition, permanent improvements in patient care become efficient resources allocated by insurers, providing better quality of service and offering the use of new technologies such as diagnostic tests and modern drugs among others, bringing about the possibility of expansion of the local market and the option of selling models of care in different regions [3]. This scenario is reflected in the management of diseases that are considered high cost and where AR is included, as the processes to access a comprehensive treatment vary depending on the provider or insurer.

The third pillar is the *quality of health care,* where efforts are focused on academic and human quality of the members of the interdisciplinary team to provide comprehensive management. The CoE should ensure that each person that is part of the path and comes into contact with patients with RA is specifically trained for this entity and know the context in which the processes are developed to avoid different obstacles. For this purpose, one of the tools used is the ongoing assessment and co-assessment to ensure competitiveness within the group, improving academics and the workplace organizational climate. Additionally, meetings or scientific committees headed by the leader rheumatologist should exalt capabilities and empower their employees to strengthen the learning curve and keep a cohesive team with permanent bargaining power. These organizational skills become important in the CoE, since they allow socializing aspects related to evidence-based medicine for joint decision making in all areas affecting the patient. On the other hand, they aim to reduce the rotation of professionals who have gained experience, improving human resource management, and directly impacting the care process, with high focus of humanism and high scientific quality [2].

Long-term consolidation of these three pillars guarantees that the learning curve increases, hence, the expertise of the interdisciplinary team provides the best care for patients with RA. In this context, clinical groups should be led by rheumatologists, and these must be accompanied by professionals in nursing, physical therapy, psychology, or psychiatry, as well as pharmaceutical chemists, social workers, and among others. Additionally, CoEs must have administrative support that enables to meet the indicators of organizational management, ensuring the administrative and logistical operation of the center (Fig. 2).

Additionally, the REAL-PANLAR group suggests a clinical overview of the characteristics of a Center of Excellence in comprehensive care in RA which supplements aspects of clinical management aforementioned by Castaño. This management must start from a medical analysis and clinical diagnosis, which helped by clinimetric results (DAS28, ACR, HAQ, CDAI, and SDAI), and diagnostic tests such as the clinical laboratory (rheumatoid factor, ACPA’s, and other labs) and conventional radiology are indispensable for the differential diagnosis, treatment, and monitoring of the patient. Once the therapy line is defined, it is vital that the patient be included in the strategy for “Treat to Target”, which seeks that the patient go towards to a proper control of RA considering the therapeutic goal clearly defined by the rheumatologist and his interdisciplinary team, involving, of course, the patient, for his active participation in this process allows for meeting the clinical objectives in place [7, 15, 16]. Associated with this, standardization of medical records management brings about as a result of the organization of information for the health professionals so that they can gain access to information regarding the different interventions performed. Parallel and from the start of treatment, patient education plays a fundamental role in management, because if the patient improves the conditions of their care, there will be a better adherence to treatment, thus improving its result. This strategy should be offered by various means (virtually, courses, and classroom lectures), after assessing patient schooling and family context for learning support [7].

### Comprehensive attention

**Fig. 4:**
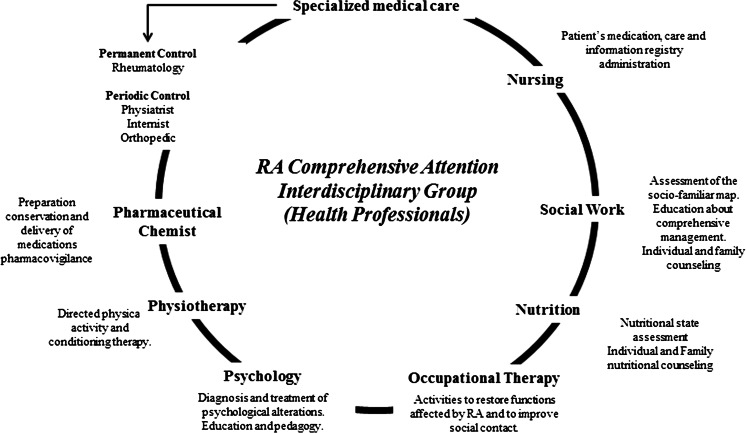
Comprehensive care for RA

Consistent with the above, a proposal for structuring a model of comprehensive care for RA is presented. This model should have as its main goal reducing the functional limitations and those related to the patient’s psychosocial performance, aiming to improve the quality of life. To meet this objective, we suggest (Fig. 4) [7, 17, 18]:

- Designing an educational program to involve the patient and to be a part of the attention process.
- Focusing efforts on the prevention of complications or avoiding disability, reducing handling costs.
- Periodic interdisciplinary control to determine disease progression and its impact.
- Evaluation of compliance to pharmacologic and non-pharmacologic treatment.
- Implementation of risk management strategies that must be cost effective for the fulfillment of therapeutic goals.

These proposals are integrated into shared responsibility, avoiding that decisions be vested in a single subject regardless of the concept of different professionals who are involved with the patient [19].

This model articulates and summarizes what was described by Li regarding the team that should take care of the patient with RA and mentioned by Castaño where the CoE is concerned.

The establishment of CoEs in RA is not limited to the administrative, but has to do with integrated health care models and the clinical ability to define and clarify the route of RA management taking into account the potential obstacles characteristic to each region.

Moreover, as already mentioned above, the CoE in its philosophy of continuous improvement must be assessed and accredited cyclically; currently, in several countries, there are accreditation bodies or health certifications for certain pathologies or all of them. In Colombia, for example, for 20 years, the Organization for Excellence in Health (Organización para la Excelencia en Salud–OES) is responsible for evaluating the Centers of Excellence in health. This organization aims to “promote and lead the improvement of health care quality through management of sector institutions” from the research, innovation, and intervention, ensuring compliance minimum standards and continuity according to the indicators established in the evaluation manual [20, 21].

This manual bases the assessment of CoEs on three items:

1. Standards
2. Evaluators
3. Evaluation and qualification process

The *standards* are based on the philosophy of continuous improvement, user-centric management, patient safety, production, and sale of services and competitiveness, within which performance measurement, health equipment, and processes of comprehensive patient care are defined. These standards must be recognized by referent experts in RA, health management, and similar RA centers of excellence, where the following parameters [17] will be considered:

- Attention based on clinical practice guidelines and experienced professionals.
- Perception of the patients and their family of the attention.
- Knowledge management should be permanent, generating research productivity, from an information system or powered by collection of patient data, ensuring confidentiality, and acting in accordance with good clinical practice standards.
- Suitable physical structure and human resource in the comprehensive care of RA.

With respect to the evaluators, they must be (1) medical professionals in rheumatology with research experience and recognition in the clinical setting and (2) health professionals expert in management and quality assessment.

Finally, the process of evaluation and assessment is based on the application of a series of checklists that assess: Verification methods in which methodology for compliance indicators and objectives defined for the CoE are included in the framework of a Manual of Evaluation [21]. Additionally, documents of constitution, minutes of committees, appointments of professional and support staff, evidence of compliance processes, and procedures in the field are reviewed. One of the medium-term proposals by the REAL-PANLAR project for the establishment of centers of excellence in RA in Latin America precisely seeks to raise the creation of a center of accreditation or certification attached to Panlar and to provide not only evaluation processes for the applicant centers but also advice on continuous improvement in the quality of care in RA [7].

As a result of the revision, we can define three factors that articulate comprehensive attention of RA, namely: the determination of a clinical route, the structure of an excellence center, and its role in patient management.

This route begins from the identification of symptoms by the patients and their consultation at the primary level, followed by referral to the rheumatologist and assessment, in order to confirm the diagnostic suspicion. This action is complemented by the interdisciplinary evaluation, which focuses on the customization of the treatment using clinical strategies and follow-up such as T2T, which involves the environment with the management, whose main goal is the patient’s global quality of life. Additionally, elements of the center of excellence are integrated to guarantee the clinical route’s quality and its timely attention by specialized professionals. These processes are evaluated continuously assuring the compliance with the indicators that give as a result the success of the medical management.

## Conclusions

RA is a prevalent and high cost disease, which needs comprehensive care by an interdisciplinary group of professionals in high-quality health, in order to provide the best care, impacting the prognosis, and involving the patient in the treatment process.

The creation of comprehensive care centers such as CoEs or similar is an initiative to continually improve processes of care and management of RA, starting from structuring scenarios that guarantee the concentration of RA patients, a team of professionals health that has expertise and humanism, leaders in innovation and research, supported by pedagogical strategies focused on the patient and under clinical monitoring of the T2T type.

This paper aims to motivate rheumatologists and scientific societies of different regions in an interdisciplinary effort to structure from centers of integrated management for RA, Centers of Excellence in RA, considering the volume of patients with this entity, the limited availability of specialized health professionals in this area and obstacles in the comprehensive care of patients.

## References

[1] Organización Panamericana de la Salud OPS (2012) Redes integradas de servicios de salud., EE.UU., http://www.paho.org/hq/index.php?option=com_content&view = category&layout = blog&id = 3184&Itemid = 3553&lang = es. Accessed 12 nov 2014

[2] Castaño R, Centros de excelencia: calidad, eficiencia y competitividad para la exportación de servicios (2011). Centros de excelencia: calidad, eficiencia y competitividad para la exportación de servicios. Rev Vía Salud.

[3] Porter M, Teisberg E (2004) Redefining Competition in Health Care. Harvard Bussines Review (2004)., June: 64-78. Available: http://ahen.ache.org/Documents/porter%20-%20Redefining%20Competition%20in%20Health%20Care.pdf. Accessed 12 dec 2014 15202288

[4] Scott DL, Wolfe F, Huizinga TW (2010). Rheumatoid arthritis. Lancet.

[5] Burgos R, Catoggio L, Galarza C, Ostojich K, Cardiel M (2013). Current therapies in rheumatoid arthritis: a Latin American perspective. Reumatol Clin.

[6] Cadena J, Cadavid M, Ocampo M, Vélez M, Anaya J (2002). Depresión y familia en pacientes con artritis reumatoide. Revista Colomb Reumatol.

[7] Santos-Moreno P, Galarza C, Caballero C, Cardiel M, Soriano E, Massardo L, et Al. (2015) REAL – PANLAR project for the implementation and accreditation of centers of excellence in rheumatoid arthritis throughout Latin America. J Clin Rheum 21(4):175–180. doi:10.1097/RHU.0000000000000247 10.1097/RHU.0000000000000247 PMC4450904 26010179

[8] Chan KW, Felson DT, Yood RA, Walker AM (1994). The lag time between onset of symptoms and diagnosis of rheumatoid arthritis. Arthritis Rheum.

[9] Ruíz A, Vidal J, Tornero J, Carbonell J, Lazaro P, Mercado D (2007). Assistance quality standards in rheumatology. Reumatol Clin.

[10] Esselens G, Westhovens R, Verschueren P (2009). Effectiveness of an integrated outpatient care programme compared with present-day standard care in early rheumatoid arthritis. Musculoskeletal Care.

[11] Li L, Badley E, MacKay C, Mosher D, Jamal S, Jones A (2008). An evidence-informed, integrated framework for rheumatoid arthritis care. Arthritis Rheum.

[12] Castillo V (2011) Centros de Excelencia y la relación docencia servicio. Centros de Excelencia y la relación docencia servicio. Primer Foro de Educación Superior en Salud en el siglo XXI, Colombia, http://www.mineducacion.gov.co/cvn/1665/articles-280558_archivo_pdf_victorcastillo.pdf. Accessed 3 oct 2014

[13] Strombeck B, Petersson IF, Vliet TP (2013). Health care quality indicators on the management of rheumatoid arthritis and osteoarthritis: a literature review. Rheumatology.

[14] Massardo L, Suárez-Almazor ME, Cardiel MH, Nava A, Levy RA, Laurindo I (2009). Management of patients with rheumatoid arthritis in Latin America: a consensus position paper from Pan-American League of Associations of Rheumatology and Latin American Group of arthritis rheumatoid study. J Clin Rheumatol.

[15] Santos-Moreno PI, de la Hoz-Valle J, Villarreal L, Palomino A, Sánchez G, Castro C (2015). Treatment of rheumatoid arthritis with methotrexate alone and in combination with other conventional DMARDs using the T2T strategy. A cohort study. Clin Rheumatol.

[16] Cardiel M (2013). Estrategia "treat to target" en la artritis reumatoide: beneficios reales. Reumatol clin.

[17] SIES Centros de excelencia Modelo de atención para artritis reumatoide (2010) Modelo de atención para artritis reumatoide Colombia. Disponible., http://www.sies.com.co/web/index.php?option=com_content&view = category&layout = blog&id = 7&Itemid = 22. Accessed 1 dec 2014

[18] Gobierno general de México (2010) Guía de práctica clínica, Diagnóstico y Tratamiento de Artritis Reumatoide del Adulto., http://www.cenetec.salud.gob.mx/descargas/gpc/CatalogoMaestro/195_ARTRITIS_REUMATOIDE /artritis_reumatoide_RR_CENETEC.pdf. Accessed 29 oct 2014

[19] Marion CE, Balfe LM (2011). Potential advantages of interprofessional care in rheumatoid arthritis. J Manag Care Pharm.

[20] Organización para la Excelencia de la Salud OES (2014) Objetivo., http://www.cgh.org.co/nosotros/nosotros.php. Accessed 30 sep 2014

[21] Organización para la excelencia en salud OES (2014) Manual de evaluación. Manual de evaluación., http://www.cgh.org.co/centrosdeexcelencia/imagenes/certificacion-manual_evaluacion.pdf. Accessed 30 sep 2014

